# Managers’ Perspectives of Quality of Care in Service Housing and Home Care Services: A Qualitative Study

**DOI:** 10.1177/23337214221142938

**Published:** 2022-12-20

**Authors:** Laura Corneliusson, Tiina Pesonen, Salla Ruotsalainen, Juhani Sulander, Anja Noro, Timo Sinervo

**Affiliations:** 1 Finnish Institute for Health and Welfare, Helsinki, Finland

**Keywords:** quality of care, older people, service housing, home care, front-line managers

## Abstract

The aim of this study was to illuminate facilitators and barriers to the quality of care in service housing and home care services, as described by managers. In total, 17 service housing and home care service front-line managers participated in this study. The interviews were conducted in Finland during October 2021 using semi-structured interviews. Qualitative content analysis was used to analyze the data. Described facilitators to the quality of care included: staff dedication and motivation, a positive psycho-social working environment, sufficient staffing, coaching management, and optimized tasks. Described barriers included: increased efficiency demands, staffing challenges, inefficient division of labor, conflicts within the working community, and disruptions due to COVID-19. The results suggest that recruiting and retaining sufficient dedicated and motivated staff is paramount to ensuring quality of care from the managerial perspective, and it seems changes in the working culture may support quality of care in a cost-efficient way.

## Key Points

- Facilitators and barriers to the quality of care from the perspective of managers seem to be interrelated, with financial resources and job strain being underlying factors influencing many facilitators and barriers.
- Recruiting and retaining sufficient dedicated and motivated staff seems to be key to ensuring quality of care within aged care services.
- Changes in the working culture, such as applying a coaching management style and task optimization, may support quality of care as well as assist in attracting and retaining staff.
- Changes in the working culture may likely require both financial investments and changes in the management and organizational systems, indicating that increased support and co-operation from the policy level may be needed to ensure quality of care.

## Introduction

The quality of care of services for older people has been widely discussed during the past years, as the number of older people is expected to globally increase (Rouzet et al., 2019; World Health Organization, 2018). In Finland, the proportion of older people (65+) is expected to increase to 26.2% of the total population by the year 2030, placing pressure on the current welfare system (Statistics Finland, 2019). Due to this anticipated demographic shift, global concerns have been voiced in relation to restricted financial resources, lack of staff within care services, and increased inequality and poverty among the older generations, which all may be seen to potentially compromise the quality of care (OECD, 2020; Rouzet et al., 2019).

In Finland, public services for older people are arranged by municipalities based on a needs assessment. Public care services for older people include around-the-clock care in long-term care facilities, service housing, and home care services in ordinary homes (Ministry of Social Affairs and Health, 2022a). In addition, older people may seek additional services from the private market at their own expense. Service housing in Finland generally provides housing with around-the-clock care of trained professionals, such as practical nurses and registered nurses, while home care services in Finland generally provides care by these same professionals at specific time points in one’s own home (Ministry of Social Affairs and Health, 2022b, 2022c). Long-term institutional care has almost been ceased (Ministry of Social Affairs and Health, 2020a). During the past years there have been considerable changes in the legislation concerning services for older people in Finland, which may be expected to influence the quality of care services. A new law on services for older people has been implemented, which will gradually increase the minimum staffing requirement in service housing facilities to 0.7 staff members per resident by the year 2023 (Ministry of Social Affairs and Health, 2020b). Furthermore, by the year 2023, having a Resident Assessment Instrument (RAI) evaluation for all older people receiving care services is to be become mandatory in Finland, which will assist in monitoring the clinical quality of care (Ministry of Social Affairs and Health, 2020b). Despite contextual differences, both home care services and service housing have the same reported objectives: to promote functional ability, independence, health, and wellbeing of older people (Ministry of Social Affairs and Health, 2020a, 2020c).

The quality of care of services for older people has often been measured by either monitoring clinical quality of care, or by surveying either staff, loved ones, or residents themselves (Kahanpää, 2019; Leppäaho et al., 2020). As older people may have physical limitations and/or cognitive impairments, proxy ratings have also been used, mainly to evaluate health-related outcomes and quality of life (Neumann et al., 2000; Robertson et al., 2020). Research on managers’ influences on, and perceptions of, quality of care in the context of care of older people is currently scarce, with many studies focusing on associations between management and leadership approaches and quality of care (Siegel & Young, 2021). A Swedish study identified that nursing home units with high degrees of person-centered care are often characterized by managers who are engaged in staff knowledge and personal development, who support providing residents with individual care, who strive to improve team spirit, and who are informed of the quality of care being provided (Backman et al., 2021). Another study from Switzerland found that managers contribute to quality of care by leading with commitment, by creating favorable conditions to high-quality care, and by continually and actively collaborating with staff, ultimately supporting a person-centered environment (Asante et al., 2021). A study on leadership style and quality of care found that a consensus leadership style, characterized by open communication between management and staff, was associated with better quality of care in the nursing home setting (Castle & Decker, 2011). There however seem to be no recent studies illuminating the specific features influencing quality of care as understood by managers.

International literature on the quality of care within services for older people from the perspective of care staff has found that quality of care often encompasses several, sometimes complex and contradictory, factors. A literature review on care work and the quality of care in older people care found a study which indicated a significant positive correlation between staff distress and the number of positive staff-resident interaction; this result may be due to these staff members empathizing with residents to a high extent (Hannan et al., 2001). This same review further indicated that management practices influence work satisfaction and the quality of care, and a recent literature review highlighted that care staff skills are an important factor in care quality (Cleland et al., 2021; Hannan et al., 2001). Study on work stressors and organizational performance in long-term care for older people in Finland has shown that work stressors, such as a high level of job demand, low levels of job control, and time pressure were associated to an increase in clinical quality of care problems (Pekkarinen, 2007). Study concerning quality of care in service housing in Finland showed that staff members that assessed the staffing of the units to be good or excellent, and who found the support of management to be good, evaluated the quality of care of residents to be high (Kahanpää, 2019.) A recent study on aspects relevant to the quality of care in home care services and service housing in Finland showed that staff report high levels of work strain, lack of support from managers and concern toward their own health and safety while working within care services for older people; staff in Finland also reported more concerns toward the quality of care and safety of the residents compared to peers in other Nordic countries (Kröger et al., 2018). It therefore seems factors influencing the quality of care of services for older people, especially from the managerial and organizational perspectives, require further attention.

Donabedian (1988, 1989) has divided quality of care into structures, processes, and outcomes. Structures refer to facilities, financing, staffing, and employees’ training, which are related to the processes. The processes, in turn are related to the ways care is delivered as well as employees relationships with clients and each other. Outcomes are related to changes in health status, patient satisfaction, or quality of life (Donabedian, 1988, 1989). The quadruple aim is a framework which aims to improve population health and health care by addressing health care systems as a whole, by focusing on four dimensions; improving population health, improving the patient experience, reducing costs, and improving the work life of the providers of services (Bodenheimer & Sinsky, 2014). Although the quadruple aim and the Donabedian model have many parallels, the quadruple aim distinctly differs from the Donabedian model by placing emphasis on staff wellbeing as a necessary element to obtain the three other aims (Bodenheimer & Sinsky, 2014; Donabedian, 1988, 1989). The quadruple aim has been widely implemented as a framework to promote the quality of health care services and has been utilized for policy development and research purposes in countries such as the United States, Canada, and Italy, to name a few (D’Alleva et al., 2019; Fitzpatrick et al., 2019; Valaitis et al., 2020). As previous studies have shown large dissatisfaction among healthcare staff concerning working conditions, the quadruple aim has been commended for including consideration toward the working conditions and needs of staff working within health care services (Fitzpatrick et al., 2019; Sikka et al., 2015; Valaitis et al., 2020).

Although the previous studies presented above have illuminated aspects relevant to quality of care from several perspectives, there seem to be no recent studies on the specific factors influencing quality of care from the perspective of managers of care services for older people. Therefore, further knowledge on the factors influencing quality of care from the perspective of managers is needed; to both better understand the current situation within care services for older people from a managerial perspective, and to assist in understanding how changes, such as in staffing, may influence the quality of care. As several countries globally, such as Japan, Germany, Italy, Korea, France, Spain, the United States, and Canada, to name a few, are also expected to face challenges in relation to the provision of care services for older people, further knowledge on factors influencing quality of care may also assist in ensuring continued quality of care (Rouzet et al., 2019). The aim of this study was therefore to illuminate facilitators and barriers to the quality of care in service housing and home care services as described by managers, and the research questions for this study were as follows:

(1) What are the perceived facilitators to the quality of care on the managerial level?
(2) What are the perceived barriers to the quality of care on the managerial level?

## Methods

### Design, Sampling, and Data Collection

This study is a part of the larger research entity the Staff Time Measurement study, which explores the availability of staff and staff time allocation according to clients’ service needs. In the Staff Time Measurement study, the units, invited on a voluntary basis, followed the working time of all their staff during 1 (service housing) or 7 days (home care). The units that participated in the Staff Time Measurement study were ones that already use the RAI-system in assessing their clients and to monitor the quality of care. The front-line managers of these units were invited to participate in this interview study by filling in their contact information. All participants who filled in their details participated in this study. A descriptive design with semi-structured interviews was used in this study. The criteria for reporting qualitative research were followed (Tong et al., 2007).

The interviews were conducted by two researchers. The participants were reminded that none of their personal information will be connected to any answers and the data will be handled confidentially. The interview guide consisted of questions on the perceived quality of care and factors relating to it, as well as questions pertaining to how care work should be developed to ensure quality of care and workers’ well-being. Follow-up questions were posed, and elaboration requested, based on the respondent’s responses. All interviews were conducted remotely using video calls during October 2021. The duration of the interviews ranged from 31 to 59 minutes. In total, 16 interviews were conducted. The final sample consisted of 10 service housing managers, one service housing assistant manager and six home care managers. The educational background of the managers varied; 10 were registered nurses, two were occupational therapists, one was a physical therapist, one was a Bachelor of Social Services, one was a Bachelor of Applied Gerontology, and one was both a registered nurse and physical therapist. All but one was female. In total, 13 managers worked in urban areas, four worked in rural areas, and the unit staff amounts varied from 11 to 50 staff members.

### Data Analysis

All interviews were audio recorded and transcribed into text. The data was analyzed using qualitative content analysis; using an inductive approach patterns and meanings were identified in the text (Graneheim et al., 2017), First, a researcher read all interviews **four** times to obtain an understanding of the data. Then, an inductive approach was adapted to identify factors which were described as facilitators or barriers to the quality of care. Factors which were described as facilitators or barriers were highlighted, and initial codes based on the contents of these statements were written in the margins of the paper. After the first initial coding of all interviews, another researcher read through the interviews and initial codes. A consensus on the contents of the interviews and initial codes was reached, and the codes were then sorted into main- and subcategories. The identified main- and subcategories were then reviewed by two other researchers who conducted the interviews, and alterations were made, until the entire research group reached a consensus concerning the main- and subcategories.

### Ethical Considerations

The study was approved by the Finnish Institute for Health and Welfare Ethical Review Board (THL/1447/ 6.02.01/2021). All participants were informed of their rights; that participation in the study is voluntary, and that they may withdraw their participation at any time without justification or consequences. The data has been stored in compliance with data regulations.

## Results

The views of managers highlighted five facilitators and five barriers to the quality of care in service housing and home care services. Described facilitators that were seen to contribute to the quality of care included: staff dedication and motivation, a positive psycho-social working environment, sufficient staffing, coaching management, and optimized tasks. Described barriers that were seen to hinder the quality of care included: increased efficiency demands, staffing challenges, inefficient division of labor, conflicts within the organization, and disruptions due to COVID-19. All facilitators and barriers were identified in both service housing and home care services, but there seemed to be some organizational variation in the results. Some organizations, both in service housing and home care, seemed to experience more issues with, for example, staffing and task division, than others (Table 1).

**Table 1.** Overview of the Results: Facilitators and Barriers to the Quality of Care in Service Housing and Home Care Services as Described by Managers.

Facilitators to the quality of care	Supportive quotation	Barriers to the quality of care	Supportive quotation
Staff dedication and motivation	“*[Having staff] with the will to better what you are doing and develop, and to make the care plans according to the rules, having a very devoted team.*”	Efficiency demands	“*One reason probably is, just that, the care needs of residents have increased. So for one resident you need more nurses and the residents are heavy, like in their physical size. For example, you need two nurses and a hoist or other tools, and the care work requires a lot, starting from basic care. Brushing teeth, helping with hygiene and other things like changing clothes for an aggressive resident can be rigid and a lot of the nurses time goes solely into the care work in my opinion.*”
A positive psycho-social working environment	“*Since the beginning I’ve felt like here they have known how to do things right, and here they really care about the staff. And on the other hand, the staff are really the type, that they do this job like it should be done.*”	Conflicts within the working community	“*People have in some sense calmed down to do their work once some people have left. That there have been those who have intentionally caused distress in the work community, I don’t know what their motives have been, but like that.*”
Sufficient staffing	“*In the recruitment, all students that are available, that have done a practical placement, we try to recruit them all, and there’s always some that stay. If a registered nurse student comes for their first practical placement, and we can get them committed, to come work for Christmas or Easter or the summer, that can help for up to four years, so that has been good.*”	Staffing challenges	“*We don’t have people to do the job, for* *example we have had no applicants for the position of a full-time nurse and the position has been vacant since last year, this is an unbelievable situation. No applicants, and now there was a practical nurse, now we have two vacancies, and a third one will open up in the end of the year, this practical nurse is changing to a completely different field. They are changing for this reason; the job is so strenuous.*”
Coaching management	“*Just like, we utilize the existing staffs’ strengths, divide tasks based on their own skills and interests. This also increases job satisfaction.*”	Disruptions due to COVID-19	“*Because the regulations changed so much in the beginning, it was hard to interpret them in the right way. And we were partially lacking safety gear in the beginning, so it was quite messy.*”
Optimized tasks	“*In this house we rotate, we strive for there not to be this is my unit- type [thinking], but instead you are a week in the one unit, and then change. The thought is that then you know all the residents and may notice in a different way when we change, that you don’t get stuck, so if a residents health shifts it doesn’t go unnoticed because you are there every day.*”	Inefficient division of labor	“*We have talked a lot, that it (the* Enterprise Resource Systems*) should divide the labor so that you just press a button, because it has the background information. So then you would only have to manually change the tasks that happen at for example a specific time. But at the moment the division of labor is a lot of, that we do a lot of manual changes. We should be able to trust the system more, that it can divide the labor.*”

### Facilitators to the Quality of Care

#### Staff dedication and motivation

Staff dedication and motivation was described as having committed and long-term staff motivated to enforce the client’s individuality, autonomy, and potential, which was described as a feature that increased quality of care. Having staff with a positive and humane approach toward care of older people was described as a feature that provided continuity and security to the clients. Having committed long-term staff enabled having assigned nurses for the clients and possibilities to update care plans, which facilitated better possibilities to provide individual support. Having dedicated and motivated staff also promoted co-operation with loved ones, working at the client’s pace, working toward fulfilling the clients wishes, and involving and activating clients, which were described as features which facilitated good quality of care. Furthermore, having engaged and committed staff and an organizational culture which reported and acted upon any potential abuse was described as a factor that promoted the rights of the clients.

#### A positive psycho-social working environment

A positive psycho-social working environment was described as having an open and supportive work community in which staff thrived, which resulted in increased quality of care. Having pleasant co-workers and support from both co-workers and managers was described as a factor that enhanced the psycho-social working climate, resulting in more content staff that provided better quality of care. Furthermore, having mutual trust between staff members and the organization, open communication and a good team spirit was described as having a positive impact on the working environment. Having enough free time to unwind and relax was also described as a factor that contributed toward a positive psycho-social working environment, as rested staff were seen to perform better.

#### Sufficient staffing

Sufficient staffing was described as having the resources to attract and retain staff, utilizing different worker groups to ensure adequate staffing, and the opportunity to adjust the number of staff to ensure good quality of care. Having the opportunities to provide different incentives to retain and attract staff, such as being able to provide permanent contracts or higher salaries was described as facilitator to good quality of care, as these organizational resources enhanced opportunities to retain motivated staff. Having the opportunity for reduced working hours, or physically easier tasks for staff with health issues was seen to ensure opportunities to sufficient staffing. Furthermore, being able to attract and hire motivated students and retired staff for part-time work was also highlighted as a factor that helped ensure adequate staffing. Having the opportunity to hire nurses’ aides was described as a factor that eased the workload of practical nurses and registered nurses. Almost 90% of employees working in care services for older people in Finland have at least 2.5 years education in health and social care, and having the opportunity to hire nurses’ aides that could assist in practical tasks was seen to free time for nurses to focus on care related matters. Having the decision-making power to adjust the number of staff, such as being able to add staff during sudden shifts in the health of the clients, was furthermore described as a feature that facilitated good quality of care.

#### Coaching management

Coaching management was described as providing staff with opportunities to autonomy, information, and further training, which was perceived to enhance the quality of care. Providing staff with sufficient information and a thorough orientation for new staff members was described as a feature that ensured adequate knowledge on the working culture and expectations. Supporting continued learning, such as providing opportunities for staff to participate in courses and learn specializations also supported professionalism. Giving staff the opportunity to participate in the planning of work, such as being able to plan their own working hours and the course of the day, was perceived as a feature which increased staff satisfaction. Furthermore, utilizing the staff’s strengths and special interests, such as when arranging activities, was also described as a feature that utilized staff competence, increased staff satisfaction, and provided clients varied activities.

#### Optimized tasks

Having optimized tasks was described as having the opportunity to utilize alternate working arrangements and specialized staff to promote both staff and client wellbeing, increasing the quality of care. Providing staff with varying tasks was understood as a feature that increased work satisfaction, as this reduced job strain by providing the staff the opportunity to care for clients requiring different amounts of care. Furthermore, in some units, job rotation and providing opportunities for permanent staff to work with different clients enabled new staff to come in and notice subtle changes in the client’s health, that risked going unnoticed otherwise. Job rotation was also seen to reduce job strain, as staff would attend to clients with varying needs. Being able to arrange the working day so that tasks are distributed not only in the morning, but throughout the day, was described as a feature that provided opportunities for clients to be assisted at their preferred times. Having well implemented digital solutions, such as an Enterprise Resource Planning (ERP) system, which is a computer-based time and task allocation system used mainly in home care services but also in service housing facilities, was furthermore described as a factor that facilitated good quality of care. The ERP systems were found to be especially beneficial when the ERP planners were staff from within the unit and who were thereby familiar with the clients and staff.

### Barriers to the Quality of Care

#### Efficiency demands

Efficiency demands were described to have resulted in increased amounts of administrative work, larger organizations, and clients having higher service needs. Increased administrative work was described as resulting in additional work and disturbances during working, with mandatory trainings, attending to documentation, and phone calls increasing job strain and reducing time doing care work. Having a large organization was described as a barrier to the quality of care, as arranging necessary services, such as maintenance work, appropriate facilities, or specialist services such as doctors’ visits or physical therapy, were perceived as difficult and limited due to having to rely on shared resources, rather than having these resources in-house. The increased care needs of clients, compared to the level of care needs exhibited during past decades, was described as a factor that resulted in increased job strain and difficulties in including the clients in activities. The decreased physical, cognitive, and psychological health of clients led to difficulties in promoting social inclusion, as well as resulted in clients requiring increased amounts of care and attention from the staff.

#### Staffing challenges

Challenges related to staffing were described as the difficulty to recruit and retain staff, which compromised the quality of care. Staff having long sick leaves and resigning in large amounts resulted in a lack of staff, while recruiting new staff was described as challenging due to the lack of applicants, the lack of language skills amongst the applicants, and the limited role of nursing assistants due to nursing assistants lacking the required license to distribute medication. In addition to the challenges in finding full-time staff, finding temporary staff to fill in for absences was also described as challenging and time consuming. Several organizations had a common internal staff pool for temporary workers, which, however, was not sufficient to cover the need for temporary staff, leading to the recruitment of external temporary workers from outside of the organization to ensure sufficient staffing. These staff members from labor hire agencies were not familiar with the clients’ and required instruction from the regular staff, which was described as a factor that compromised the quality of care. Described reasons behind the difficulty to recruit and retain staff were the low salary of staff in relation to the amount of responsibility their profession requires, the large amount of job strain, as well as the overall negative perceptions toward care work. However, the challenges in obtaining staff varied, with some managers reporting little to no difficulty in obtaining staff; this was perceived to be due to the good reputation of the organization.

#### Inefficient division of labor

The inefficient division of labor due to traditional working hours and problems with the ERP were described as factors that hindered the possibilities to do good quality work and provide good quality care. The traditional division of working hours, with the focus of activities being during the morning hours, was perceived as a barrier to the quality of care as this resulted in hectic morning shifts, and clients having to adapt to the staffs’ schedules. Problems related to the ERP included the systems inability to recognize the clients’ assigned nurses, difficulties in finding the necessary information in the system, communication issues between the staff and ERP planners when the planning was centralized, and the sometimes poor division of labor. These challenges with the ERP resulted in unnecessary travel or additional labor, which reduced the amount of time doing care work.

#### Conflicts within the working community

Conflicts within the working community were described as communication issues and lack of motivation among staff, which were perceived as barriers to the quality of care. Due to communication and trust issues between staff and middle and upper management, suggestions and proposal to improve the quality of care were perceived as hard to implement, and lack of trust and disagreements amongst the staff led to conflicts that reduced staff wellbeing. Staff that were perceived as not motivated for working in care services were also described as barriers to the quality of care, as non-motivated staff were described as causing conflicts within the working community, as well as delivering lower quality of care. Staff from labor hire agencies having higher salaries than permanent and part-time in-house employees was also described as a factor that caused friction within the working community, as these workers would boast about their higher salary.

#### Disruptions due to COVID-19

Disruptions due to COVID-19 were described as decreased opportunities to social interaction, changing guidelines, issues related to safety gear, and staff absences which compromised the quality of care. Staff absences were described as resulting in lower quality of care, as replacement staff were difficult to obtain, and activities for the clients needed to be cancelled. Reduced social interaction due to closed units and restrictions was described as having impacted both the staff and clients. Reduced social interaction between co-workers was described as a feature which decreased work satisfaction, while reduced social interaction with and between the clients, staff, and loved ones resulted in loneliness amongst clients. In the beginning of the pandemic, lacking safety gear caused concerns, while during the continuation of the pandemic having to work in protective safety gear was uncomfortable for staff, decreasing staff satisfaction.

## Discussion

The results of this study show that the aspects related to quality of care from the perspective of managers seem to be interrelated; some facilitators to the quality of care could also be seen as barriers. For instance, while staff employed from labor hire agencies helped ensure sufficient staffing, they could also compromise the quality of care. Staff from hire labor agencies were generally unfamiliar with the clients, required instructions and direction from the permanent staff, and could also cause conflicts within the working community. Previous study has also shown that permanent staff members that work in organizations that use staff from labor hire agencies find they are less able to develop professional skills, and experience increased job intensity and unequal treatment of employees (Saksanen et al., 2022). These results indicate that to ensure quality care, special attention should be paid on how to retain dedicated and motivated staff. Other studies from the United States, Australia, and the Netherlands have found that staff attributes and stability seem to be significant factors contributing to the quality of care; higher qualification among nurses has been associated with higher care quality in nursing home context (Hyer et al., 2011; Spilsbury et al., 2011), and staff stability has been found to be an important factor in better care quality (Castle & Engberg, 2007). In this study, the managers described having possibilities to better answer the clients’ individual needs if the staff was familiar with the clients, but also described job rotation as a factor that enabled balancing the workload. Interestingly, merely having adequate staffing was also described by some as a facilitator to quality of care. Although hiring staff from labor hire agencies could compromise the quality of care, having staff present is a prerequisite for care to take place. Previous studies have found that having adequate staffing may increase quality of care when examining clinical outcomes; however, it has been argued that quality of care often relies on indicators that are easy to measure rather than inclusion of aspects relevant for all stakeholders (Backhaus et al., 2014; Spilsbury et al., 2011). The results of this study seem to suggest that striving toward quality of care on specifically the managerial level may currently be an interplay between financial resources and the often immediate demand for staff. It also seems that in the absence of long-term financial resources to invest in staff, quality of care on the managerial level may need to be viewed as equivalent to quantity of staff. Thereby, developing measures to attract and retain dedicated and motivated staff within aged care services seems to be paramount while striving to ensure quality of care.

Working conditions, job satisfaction, and staff wellbeing have been shown to be pertinent to recruiting and retaining staff within health care services (Jones, 2017). An argument for including staff wellbeing in the quadruple aim has been studies which indicate decreasing staff wellbeing over the past years (Sikka et al., 2015). This decrease in staff wellbeing is speculated to be due to increased efficiency and productivity demands, resulting in, for example, increased non-care related work, dysfunctional working environments, disrespect between staff and lack of work engagement (Sikka et al., 2015). The results of this study seem to corroborate increased efficiency demands as a factor influencing work engagement, and the interrelated nature of the results of this study, such as the challenges associated to staffing, illuminate the significance of financial resources and working conditions to the actualization of quality care. Previous studies have also highlighted factors such as work stressors, work strain, number of staff, and support of management being influential to the quality of care (Kahanpää, 2019; Kröger et al., 2018; Pekkarinen, 2007). While the results of this study highlighted incentives such as higher salaries and alternate working arrangements as factors that helped ensure adequate staffing, previous studies have indicated organizational factors, such as a supportive working culture, as important factors to retaining and recruiting staff (Jones, 2017). It therefore seems addressing issues associated to retaining and maintaining sufficient motivated and dedicated staff may require interventions on the managerial, organizational, and potentially even policy level, as financial resources may be needed to make organizational changes, hire more staff, or increase salaries.

The results of this study suggest that striving toward quality of care requires a process of compromise and balance between the quadruple aims, which has also been illuminated in previous studies (Arnetz et al., 2020; Bachynsky, 2019). Interestingly, a smaller pilot study has suggested that increasing efficiency by utilizing staff strengths and adjusting work processes may support workplace wellbeing and patient experiences (Arnetz et al., 2020), and factors such as task optimization and adapting a coaching management style were described as facilitators to quality of care in this study as well. These results suggest that adapting a coaching management style, which may be described as a management style that aims to involve and empower staff, and changes in the working culture and working arrangements may support quality of care in a cost-efficient way (Evered & Selman, 1989). A previous study has found that the management systems within care services for older people may often consist of non-democratic leadership and hierarchical power dynamics (Syvänen, 2003). This same study also found that short-sighted aspirations toward cost-efficiency by implementing staff cut-backs may result in high organizational inefficiency, resulting in difficulties to implement change (Syvänen, 2003). Changes in the working culture may therefore likely require both financial investments and changes in the management and organizational systems, indicating that increased support and co-operation from the policy level may be needed to ensure quality of care. Future studies may seek to explore if, and how, changes in working arrangements and the working culture support quality of care, and what resources are required to obtain these changes. Furthermore, as task division and working conditions seem to be an influential factor to quality of care, the use of Enterprise Resource Planning (ERP) systems seems to require further attention. Previous studies have shown that within aged care services the use of ERP systems has been described as resulting in an inefficient division of labor, less time to provide care work, and increased stress (Bordi, 2019; Rytkönen, 2018). However, the results of this study indicated that the ERP system may be both a barrier and facilitator to the quality of care; having staff that were familiar with the clients plan the ERP task division was perceived as a facilitator to the quality of care, and previous study has found that having familiar staff plan the ERP task division increased efficiency and enabled staff to spend more time with clients (Noro & Karppanen, 2019). Better distribution of resources within home care services has also been shown to be able to both reduce costs and enable a more efficient distribution of workload among staff (Groop, 2012). Therefore, further research into the ERP systems in aged care may be needed to ensure that resources are being allocated appropriately.

Although these results illuminate many factors relevant to the quality of care in service housing and home care services from the perspective of managers, it seems more knowledge is needed on how quality of care can be best sustained while needing to balance between restricted resources and ensuring staff wellbeing. While the results suggest that a coaching management style, with a focus on alternate working arrangements and changes in the working culture may support quality of care, financial resources and organizational structures may influence the extent to which changes may be made. Therefore, further research seems to be needed within how to recruit and retain motivated and dedicated staff in a financially sustainable way; specifically, if and how changes in the working culture and working arrangements may assist in retaining staff. Future studies within the staff time measurement project will seek to explore these questions.

## Conclusions

The results of this study illuminate how the facilitators and barriers to quality of care from the perspective of managers seem to be interrelated, with financial resources and job strain being underlying factors influencing many facilitators and barriers. The results suggest that recruiting and retaining sufficient dedicated and motivated staff is paramount to ensuring quality of care, and it seems alternate working arrangements and changes in the working culture may assist in attracting and retaining staff and support quality of care. However, further studies are still needed to understand how quality of care can be best supported while facing restricted financial resources. These results may assist in assessing which policy and practice interventions are needed to facilitate continued quality of care, both in Finland and globally.

## Limitations

One limitation of this study is the limited generalizability of the results. Due to international differences in aged care systems, these results may not be generalizable to other cultural and political contexts. Although the sample included managers from a large geographical area in Finland, and included both public and private services, it is still possible that the results are not completely representative of all service housings and home care services. Due to the qualitative nature of this study, it is possible that the analysis was influenced by the backgrounds of the researchers; however, the researchers represent different academic backgrounds, and the process has been described in detail, potentially reducing this risk.
